# High Catalytic Performance of Mn-Doped Ce-Zr Catalysts for Chlorobenzene Elimination

**DOI:** 10.3390/nano9050675

**Published:** 2019-05-01

**Authors:** Lei Zhu, Xi Li, Zhiying Liu, Lin Yao, Peng Yu, Ping Wei, Yanhua Xu, Xingmao Jiang

**Affiliations:** 1 College of Biotechnology and Pharmaceutical Engineering, Nanjing Tech University, Nanjing 210009, China; zhul_88@163.com (L.Z.); weiping@njtech.edu.cn (P.W.); 2 School of Environmental Science and Engineering, Nanjing Tech University, Nanjing 210009, China; lixi_26@163.com (X.L.); njtyaolin@163.com (L.Y.); yspong@163.com (P.Y.); 3 National Supervision and Testing Center of Fine Chemicals, Taizhou 225300, China; 4 School of Chemical Engineering and Pharmacy, Wuhan Institute of Technology, Wuhan 430205, China; jxm@wit.edu.cn

**Keywords:** MnOx-CeO_2_-ZrO_2_, catalytic combustion, chlorobenzene, active oxygen, product analysis

## Abstract

Mn-Ce-Zr-O catalysts doped with varying Mn content were prepared and assessed for the catalytic combustion of chlorobenzene (CB). Nanosized MCZ-0.67 catalyst with amorphous phase exhibited a high and stable catalytic activity among the studied catalysts, achieving 90% CB conversion at 226 °C and withstanding stability tests, including time-based stability and the successive influence of various operating conditions. Meanwhile, the MCZ-0.67 catalyst used showed good recyclability by calcination in air. Characterization results suggested that Mn doping played a dominant role in improving the catalytic performance, resulting in larger surface area, better redox properties and greater amounts of surface active oxygen. In addition, the introduction of Zr was also indispensable for maintaining the good catalytic performance of catalysts. Finally, trace amounts of polychlorinated by-products during CB oxidation were monitored and the oxidation process was discussed.

## 1. Introduction

Chlorinated volatile organic compounds (CVOCs), widely used as solvents, additives and raw materials in industry, are considered serious hazards to human health and the environment owing to their high toxicity, volatility and durability [1,2]. It is therefore of great significance to effectively control the environmental emissions of these compounds. Compared with conventional thermal combustion (operating above 1000 °C), catalytic combustion is a promising heterogeneous catalytic oxidation technology for the elimination of CVOCs due to its much lower energy consumption (operating below 550 °C) and limited secondary pollution (avoiding the production of NO_x_ and dioxins) [3]. However, the development of this technology is dependent on the advent of high-performance catalysts, which are regarded as the crucial enablers in most chemical reaction processes [4].

To date, despite much research on various types of catalysts, including supported noble metals [5,6], transition metal oxides [7,8] and zeolites [9,10] for CVOCs elimination, the occurrence of chlorine poisoning, the mass production of polychlorinated by-products and the low selectivity of HCl remain “bottlenecks” for the practical application of such catalysts. Limited natural resources and their susceptibility to chlorine poisoning restrict the widespread use of noble metals [11] despite their excellent activity and selectivity. Similarly, coking and chlorination reactions readily occur in zeolite catalytic systems [7,12]. Conversely, transition metal oxides possess a better thermal stability, stronger anti-poisoning ability and lower cost, thus arousing widespread interest [13,14]. In recent years, ceria has been used as a good promoter for the activity and structure of transition metal oxides owing to its high oxygen storage capacity (OSC) and relatively easy redox cycle between Ce^4+^ and Ce^3+^ [15,16]. Specifically, the redox capability and dispersity of transition metal oxides are modified along with the partial substitution of Ce^4+^ in the CeO_2_ lattice with metal ions. The resulting improvements in oxygen vacancy concentration and oxygen mobility contribute to the elimination of adsorbed chlorine species, and thus to the enhancement of catalytic activity and stability for CVOCs elimination [17].

Of the Ce-based transition metal oxides, a CeO_2_-ZrO_2_ solid solution, widely applied in the purification of vehicle exhaust [18], the selective catalytic reduction (SCR) of NO_x_ [19] and the catalytic oxidation of VOCs [20], was found to exhibit a high OSC and thermal stability after the doping of a proper amount of ZrO_2_ into the CeO_2_ lattice. Gutiérrez-Ortiz et al. [21] prepared a series of Ce_x_Zr_1−x_O_2_ solid solutions for the elimination of chlorinated aliphatic hydrocarbons, and showed that the catalytic performance was dominated by the increased surface acidity and oxygen mobility following Zr doping. However, considerable amounts of Cl_2_ resulting from the oxidation of HCl (Deacon reaction) were detected during the oxidation of chlorinated reactants. Moreover, due to the relatively low redox ability of ZrO_2_, the high reaction temperature required for the elimination of CVOCs by CeO_2_-ZrO_2_ mixed oxide catalysts was unsatisfactory [22].

MnO_x_ compounds are highly active due to their various labile oxygen species and high efficiency in reaction/oxidation cycles [23] and have been widely used in the catalytic removal of various pollutants, including benzene series [24], chlorinated aromatic hydrocarbons [25], nitrogen oxides [26] and aldehydes [27]. In particular, MnO_x_-CeO_2_ solid solution, as an environment-friendly catalyst with high catalytic efficiency, has become the focus of research on multipurpose catalysts [28]. Nevertheless, in the elimination of CVOCs, despite the activity and chlorine resistance of CeO_2_ having been greatly improved by Mn doping, the achievement of a high and stable catalytic activity still requires higher reaction temperature [29,30]. In addition, trace amounts of polychlorinated by-products resulting from the incomplete oxidation of CVOCs need to be further monitored and assessed.

Considering the respective advantages of MnO_x_ and CeO_2_-ZrO_2_ catalysts, Mn-Ce-Zr-O ternary catalyst seems to have promising prospects in catalysis. Shen et al. [19] prepared a series of Mn/Ce-Zr-based catalysts for the SCR of NO. Results showed that the molar ratio of Ce to Zr had a significant influence on the catalytic activity, and MnO_x_/Ce_0.5_Zr_0.5_O_2_ catalyst possessed the best NO conversion. However, the influence of Mn doping on the catalytic performance of CeO_2_-ZrO_2_ catalysts has seldom been systematically studied, especially for the elimination of CVOCs. Herein, Mn-Ce-Zr-O catalysts doped with a varying Mn content were synthesized and used for the elimination of CB, which is a typical CVOCs model widely used in the evaluation of catalysts [31,32]. The physicochemical properties of the prepared catalysts were systematically assessed by various material characterization techniques. The successive influence of CB concentration, space velocity and water vapor on CB destruction was also assessed in the stability study. Moreover, trace amounts of organic by-products during CB oxidation were monitored and the oxidation process was discussed.

## 2. Materials and Methods

### 2.1. Catalyst Preparation

Mn-Ce-Zr-O catalysts were synthesized by the sol-gel method as follows: stoichiometric amounts of Mn(NO_3_)_2_ (SCRC, 50 wt% solution), Ce(NO_3_)_3_·6H_2_O (SCRC, 99%), ZrO(NO_3_)_2_·5H_2_O (Aladdin, 99.5%) and citric acid (SCRC, 99.5%, citric acid/(Mn + Ce + Zr) = 0.3, molar ratio) were dissolved in deionized water and slowly heated to 80 °C, maintaining this temperature with continuous vigorous stirring until gelation occurred. The obtained gel was dried overnight at 110 °C and then calcined in a muffle furnace at 200 °C for 2 h and further at 550 °C for 5 h. The synthesized catalysts were denoted as MCZ-x (x = 0, 0.33, 0.50, 0.67 and 0.80), where x is the molar ratio of Mn/(Mn + Ce + Zr) and the molar ratio of Ce/Zr was maintained as 1. As a reference, a Mn_0.67_Ce_0.33_ catalyst (denoted as MC-0.67) was also prepared by the same method described above. Before use, all synthesized catalysts were finally made into 40–60 mesh particles through pressing, crushing and sieving.

### 2.2. Catalyst Characterization

Powder X-ray diffraction (XRD) patterns were recorded on a D8-ADVANCE diffractometer (Bruker, Karlsruhe, Germany) equipped with Cu Kα radiation source (λ = 0.15418 nm). The 2θ was measured from 20° to 80° at a scanning rate of 0.04 ° min^−1^. 

N_2_ adsorption-desorption experiments were carried out on a Tristar II 3020 instrument (Micromeritics, Atlanta, USA) at −196 °C. Prior to measurements, catalysts were degassed at 200 °C under vacuum for 2 h. The Brunauer–Emmett–Teller (BET) and Barrett–Joyner–Halenda (BJH) methods were employed for the calculation of specific surface area and pore-size distribution, respectively.

H_2_-temperature programmed reduction (H_2_-TPR) tests were measured under a mixed stream of H_2_ (10 vol%) and Ar (90 vol%), and 100 mg of the catalyst was tested in the range of 50–800 °C with a heating ramp of 10 °C min^−1^. During the tests, a thermal conductivity detector (TCD) was employed to monitor the consumed H_2_.

X-ray photoelectron spectroscopy (XPS) measurements were carried out on a PHI 5000 VersaProbe instrument (Ulvac-Phi, Chigasaki, Japan) equipped with Al Kα (hv = 1486.6 eV) radiation source. Binding energies of the studied atoms were calibrated by 284.6 eV of C 1s. The obtained spectra were deconvoluted by peak fitting using the Shirley background and Gaussian–Lorentzian function provided by the XPSPeak software.

O_2_-temperature programmed desorption (O_2_-TPD) tests were carried out on an Auto Chem II 2920 instrument (Micromeritics, Atlanta, USA), and 100 mg of the catalyst was tested in the range of 50–700 °C under a He stream at a heating ramp of 10 °C min^−1^. During the tests, a TCD detector was employed to monitor the desorbed O_2_. Prior to measurements, catalysts were treated at 300 °C for 2 h under a He stream and then at 50 °C for 2 h under a mixed stream of O_2_ (5 vol%) and He (95 vol%).

Scanning electron microscopy (SEM) images were taken on a Hitachi S4800 apparatus.

Transmission electron microscopy (TEM) and scanning transmission electron microscopy (STEM) images were taken at a voltage of 200 kV on a JEM-2100F microscope (JEOL, Akishima, Japan) equipped with an energy dispersive X-ray spectrometer (EDX). Powder catalysts were previously treated by dispersing them in ethanol and then depositing on carbon-coated copper grid.

### 2.3. Catalytic Performance Evaluation

Activity tests were carried out on a conventional fixed-bed microreactor. In each test, 200 mg of the catalyst was packed in the middle of the quartz tube (4 mm i.d.). N_2_ was used to transport the CB vapor by bubbling the CB solution maintained at 40 °C. The reaction gas, consisting of 1000 ppm CB and 21 vol% O_2_, was obtained by mixing three gas streams of CB vapor, O_2_ and N_2_ (balance) in a mixer. All gas streams were accurately monitored by mass flowmeters and the gas hourly space velocity (GHSV) was set at a typical value of 20,000 h^−1^. A thermocouple was employed to control the reaction temperature in the range of 100–300 °C. At given temperatures, the exhaust gases were detected on-line using a gas chromatograph (FULI 9790) equipped with two flame ionization detectors (FID), one of which was used for the analysis of CB quantitatively and the other for the measurement of CO_2_ and CO by means of a methane-converting furnace (Ni catalyst). The concentrations of HCl and Cl_2_ were determined by bubbling the off-gas through a 0.0125 mol L^−1^ NaOH solution for 30 min. Ion chromatograph (Thermo Scientific ICS-900) was used to measure the concentration of Cl^−^ and chemical titration with ferrous ammonium sulphate (FAS) using *N*,*N*-diethyl-p-phenylenediamine (DPD) as an indicator was employed to determine the concentration of Cl_2_ in the bubbled solution.

The stability experiments of the prepared catalysts were performed at 300 °C under the same evaluation conditions described above. The water vapor environment was obtained by bubbling the deionized water with a N_2_ stream.

Assessment of the CB decomposition products was performed by using hexane to absorb the various gaseous organics in the off-gas for 30 min. After concentration, the hexane was analyzed by a gas chromatography-mass spectrometer (GC-MS, Agilent 7890B-5977A) using a weak polar DB-5MS capillary column.

## 3. Results and Discussion

### 3.1. X-ray Diffraction (XRD) Characterization and N_2_ Adsorption-Desorption Analysis

**Figure 1:**
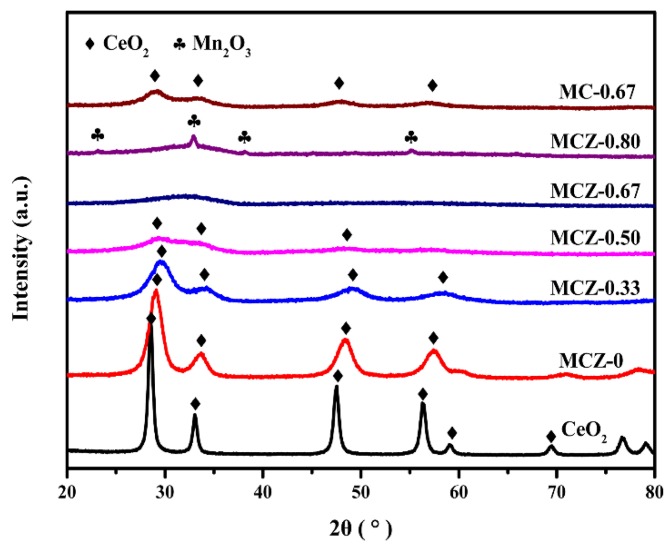
X-ray diffraction (XRD) patterns of MCZ-x (x = 0, 0.33, 0.50, 0.67, 0.80) and MC-0.67 catalysts.

XRD patterns of MCZ-x and MC-0.67 catalysts showed that the crystallinity of the prepared catalysts was significantly affected by Mn content (Figure 1). For the MCZ-x (x = 0, 0.33, 0.50) and MC-0.67 catalysts, only diffraction peaks corresponding to the cubic fluorite-like structure of CeO_2_ were observed, with the lattice parameters (Table 1) being smaller than 5.41 Å of pure CeO_2_ (JCPDS #43-1002). Indeed, the ionic radii of Mn^n+^ (Mn^2+^ = 0.083 nm, Mn^3+^ = 0.065 nm, Mn^4+^ = 0.053 nm) and Zr^4+^ (0.084 nm) are both smaller than that of Ce^4+^ (0.097 nm) [29,33]. Thus, the shrinkage of CeO_2_ unit cell indicates that parts of Ce^4+^ ions in CeO_2_ lattice were replaced by Mn^n+^ and Zr^4+^ ions to form a solid solution. Remaining Mn- and Zr-related oxides may be uniformly distributed on the surface of CeO_2_ in a poor crystalline state. At the 2θ range from 24° to 40°, only a weak and widened peak was observed for the MCZ-0.67 catalyst, indicating that it exists in an amorphous state. With the further increase of Mn content, weak diffraction peaks indexed to Mn_2_O_3_ (JCPDS #24-0508) were observed for the MCZ-0.80 catalyst, showing that the Mn content has exceeded the maximum that the CeO_2_ lattice can hold and therefore Mn_2_O_3_ phase is formed during calcination.

As is known, a lower intensity diffraction peak indicates a reduction in crystallinity and reveals the generation of more lattice defects and smaller crystallite sizes [29,34]. Thus, from Figure 1, it can be inferred that the CeO_2_ lattice is constantly distorted with increasing Mn doping, resulting in a decrease in crystallite size (Table 1). Similarly, the specific surface area and pore volume increase, reaching a maximum when x = 0.67. This observation may be attributed to the production of smaller nanoparticles with an amorphous phase. Further, the addition of Zr promotes the transformation of the MC-0.67 catalyst from a solid solution to an amorphous material and leads to a doubling of the specific surface area. This significant increase may be related to the larger solubility of Zr (maximum of 75 at%) in the CeO_2_ lattice than that of Mn (maximum of 20 at%) [35], as improved solubility can lead to the formation of more lattice defects, which is beneficial for the increase of specific surface area.

**Figure 2:**
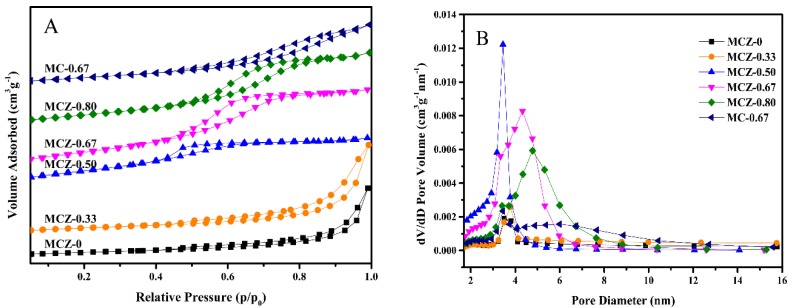
N_2_ adsorption-desorption isotherms (**A**) and pore size distributions (**B**) of MCZ-x (x = 0, 0.33, 0.50, 0.67, 0.80) and MC-0.67 catalysts.

With regards to N_2_ adsorption-desorption and pore size distribution, all catalysts prepared by the sol-gel method showed a typical irreversible type IV isotherm (Figure 2A), with the MCZ-0 and MCZ-0.33 catalysts displaying a hysteresis loop of type H3 and the remaining four catalysts displaying a hysteresis loop of type H2. Furthermore, the sizes of the formed pores were within the range of 2 to 10 nm (Figure 2B and Table 1), suggesting the presence of a mesoporous structure.

### 3.2. H_2_-Temperature Programmed Reduction (H_2_-TPR) Characterization

**Figure 3:**
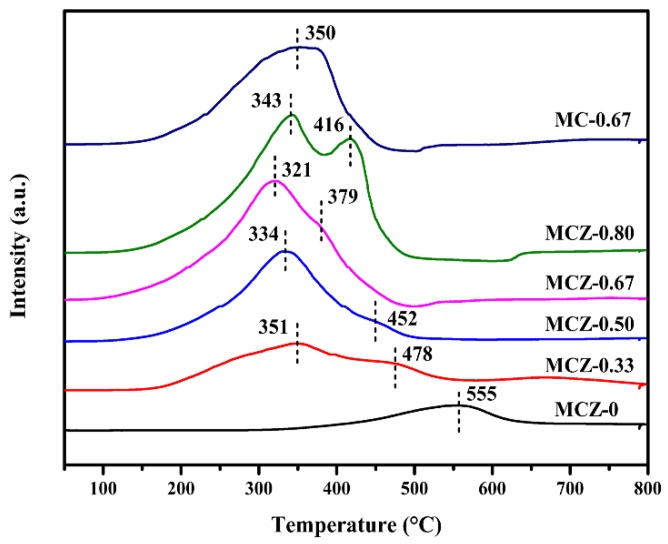
H_2_-TPR profiles of MCZ-x (x = 0, 0.33, 0.50, 0.67, 0.80) and MC-0.67 catalysts.

The influence of Mn doping on the redox properties of the catalysts was studied using H_2_-TPR (Figure 3). For the MCZ-0 catalyst, just one reduction peak appeared at 555 °C, ascribed to the reduction of Ce^4+^ on the catalyst surface [20] due to the difficulty of Zr^4+^ reduction at temperatures below 900 °C under this test condition [19]. After Mn doping, two overlapped reduction peaks at 351 °C and 478 °C were observed from the MCZ-0.33 catalyst, ascribed to the two-step reduction of MnO_2_/Mn_2_O_3_ leading to the sequential formation of Mn_3_O_4_ and MnO [36]. Similarly, with the increase of Mn content, the reduction peaks of Mn oxides moved towards lower temperatures, with the lowest reduction temperature of 321 °C being observed for the MCZ-0.67 catalyst. This was attributed to the generation of more lattice defects following Mn doping, which is conducive to the mobility of oxygen species and thus makes the catalyst more prone to reduction.

Additionally, the introduction of Mn significantly increased the total H_2_ consumption, from 0.834 mmol g^−1^ for the MCZ-0 catalyst to 6.151 mmol g^−1^ for the MCZ-0.80 catalyst (Table 1), indicating that Mn oxides play a crucial role in the reducibility of MCZ-x catalysts. Nevertheless, despite an equal Mn content, the total H_2_ consumption of MC-0.67 catalyst (4.334 mmol g^−1^) was smaller than that of MCZ-0.67 catalyst (5.081 mmol g^−1^), suggesting that the introduction of Zr contributes to the generation of more reactive oxygen. It is generally acknowledged that a lower reduction temperature shows better redox properties and that greater H_2_ consumption improves the catalytic oxidation activity. Therefore, it can be predicted that MCZ-x catalysts with a high Mn content should have a good catalytic performance in CB elimination.

### 3.3. X-ray Photoelectron Spectroscopy (XPS) Characterization

**Figure 4:**
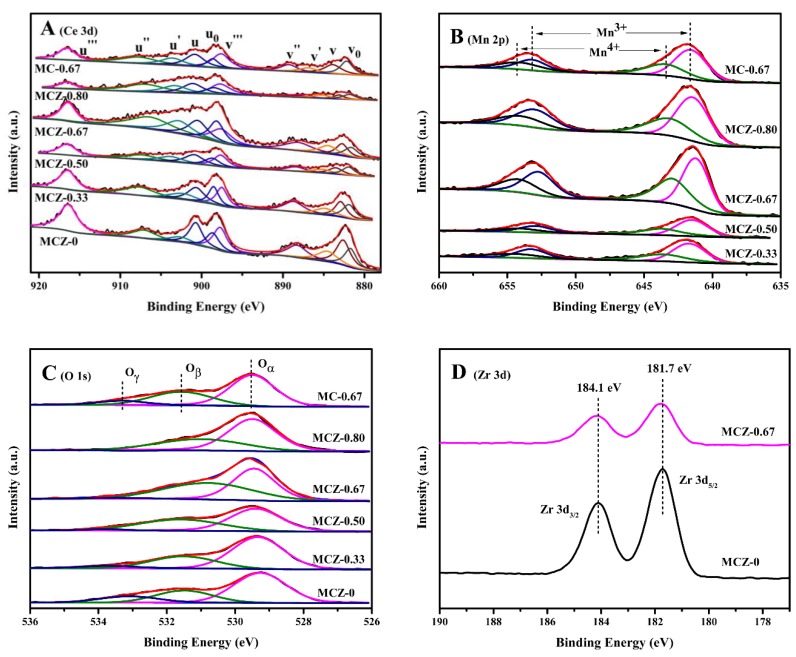
X-ray photoelectron spectroscopy (XPS) spectra of Ce 3d (**A**); Mn 2p (**B**); O 1s (**C**) and Zr 3d (**D**) of MCZ-x (x = 0, 0.33, 0.50, 0.67, 0.80) and MC-0.67 catalysts.

The surface characteristics of the synthesized catalysts were analyzed using XPS measurements (Figure 4 and Table 2). The Ce 3d XPS spectra were fitted and deconvolved into 10 peaks (Figure 4A), wherein those labeled as v, v′′, v′′′, u, u′′ and u′′′ were ascribed to Ce^4+^ and those labeled as v′, v_0_, u′ and u_0_ were ascribed to Ce^3+^ [37]. These peaks provided evidence of the coexistence of Ce^4+^ and Ce^3+^ on the catalysts surface. With the increase of Mn content, the percentage of Ce^3+^/(Ce^3+^ + Ce^4+^) increased firstly and then decreased, reaching a maximum value of 34.3% for the MCZ-0.67 catalyst. In contrast, the Ce^3+^ concentration on the MC-0.67 catalyst surface was 31.3%; therefore, the introduction of Zr promoted the transformation of Ce^4+^ to Ce^3+^. As previously reported, the presence of Ce^3+^ ions indicates the generation of oxygen vacancy and creates a charge imbalance [38]; therefore, for maintaining electrical neutrality within the catalyst, more active oxygen species (i.e., O^2−^, O_2_^2−^, O^−^) will be generated on the catalyst surface. Thus, it can be inferred that a catalyst with a higher percentage of Ce^3+^/(Ce^3+^ + Ce^4+^) could promote the rapid elimination of CB effectively because of the greater number of active oxygen species.

The measured Mn 2p XPS spectra showed four peaks fitted from the spin-orbit doublet of Mn 2p_3/2_ and Mn 2p_1/2_ at approximately 641, 653 eV and 643, 654 eV (Figure 4B), ascribed to Mn^3+^ and Mn^4+^ species on catalyst surface, respectively [39]. The actual molar ratio of Mn/(Mn + Ce + Zr) measured by XPS was close to the theoretical value (Table 2), indicating that Mn oxides are uniformly distributed on the surface and bulk of the prepared catalysts. It is noteworthy that the increase of Mn content is accompanied by the simultaneous increase in Mn^4+^/(Mn^3+^ + Mn^4+^) and Ce^3+^/(Ce^3+^ + Ce^4+^) percentages, except for MCZ-0.80 catalyst, indicating that the doping of Mn could promote the electron transfer from Mn^3+^ to Ce^4+^ on the catalyst surface. As previously reported, more Mn^4+^ ions could offer more sufficient active sites during the CB oxidation, since the oxidation reaction usually takes place through the redox cycles between high and low valence cations [40]. For the MCZ-0.80 catalyst, the lower percentage of Mn^4+^/(Mn^3+^ + Mn^4+^) may be attributed to the formation of more Mn_2_O_3_ phase during the calcination process, as evidenced by XRD characterization.

The O 1s XPS spectra obtained were fitted into three peaks (Figure 4C), with those at approximately 529.3–529.5 eV being ascribed to lattice oxygen (O^2−^, labeled as O_α_), and those at approximately 530.8–531.6 eV and above 533.0 eV being ascribed to surface chemisorbed oxygen (O^2−^ and O^−^, labeled as O_β_) and hydroxyl species and/or chemisorbed water species (labeled as O_γ_), respectively [41]. It can be observed that the percentage of O_β_/(O_α_ + O_β_ + O_γ_) experiences a similar variation tendency to that of Ce^3+^ concentration as a result of Mn doping (Table 2), confirming the analysis of surface active oxygen species in Ce 3d XPS characterization. As is known, surface chemisorbed oxygen is vital for most catalytic oxidation reactions [33,42]. Therefore, compared with the other studied catalysts herein, the high O_β_ concentration on the MCZ-0.67 catalyst surface could be responsible for the enhancement in catalytic performance for CB elimination. Likewise, the introduction of Zr could likely promote the generation of more chemisorbed oxygen on the MCZ-0.67 catalyst surface compared to that on the MC-0.67 catalyst surface (Table 2), in agreement with TPR characterization.

The Zr 3d XPS spectra of MCZ-0 and MCZ-0.67 catalysts exhibited two peaks at 181.7 eV and 184.1 eV (Figure 4D), ascribed to Zr 3d_5/2_ and Zr 3d_3/2_, respectively [43], confirming the solo presence of Zr^4+^ ions in the prepared catalysts [42]. As can be observed, for MCZ-0 and MCZ-0.67 catalysts, the binding energy position of Zr is highly similar, indicating that the chemical state of Zr is stable and hardly affected by the introduction of Mn.

### 3.4. O_2_-Temperature Programmed Desorption (O_2_-TPD) Characterization

**Figure 5:**
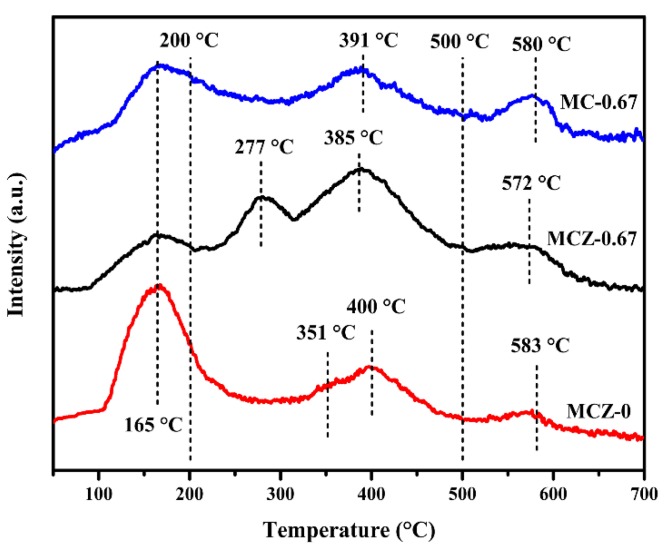
O_2_-temperature programmed desorption (O_2_-TPD) patterns of MCZ-0, MCZ-0.67 and MC-0.67 catalysts.

The distribution of oxygen species was also explored by O_2_-TPD for the MCZ-0, MCZ-0.67 and MC-0.67 catalysts (Figure 5). The desorption peak below 200 °C was attributed to physically adsorbed oxygen molecule (O_2_), while the desorption peaks within 200–500 °C were ascribed to chemisorbed oxygen species (O^2−^ and O^−^, where O^2−^ is easier to desorb than O^−^). The desorption peak observed above 500 °C was attributed to surface lattice oxygen (O^2−^) [41].

For the MCZ-0 catalyst specifically, four peaks were observed at 165 °C, 351 °C, 400 °C and 583 °C, corresponding to the desorption of O_2_, O_2_^−^, O^−^ and O^2−^, respectively. The MCZ-0.67 catalyst presented similar desorption peaks, yet with a much greater peak intensity of chemisorbed oxygen species, indicating that the addition of Mn contributes to the adsorption of active oxygen species on the catalyst surface. This is in good agreement with the O 1s XPS characterization, and likely also correlated to the large BET and mesoporous structure of the MCZ-0.67 catalyst, which are conducive to the adsorption and activation of gas-phase oxygen on the catalyst surface. The desorption peaks of the MCZ-0.67 catalyst showed a shift towards lower temperatures as compared with the MCZ-0 catalyst. As is known, a larger peak intensity and lower desorption temperature correspond to improved catalytic activity [44], which perhaps forecast the better catalytic performance of the MCZ-0.67 catalyst for CB elimination. Finally, the MC-0.67 catalyst exhibited three distinct desorption peaks; it is likely that the O_2_^−^ desorption peak may have been covered by adjacent peaks. As compared with the MCZ-0.67 catalyst, it is obvious that the addition of Zr contributes to the improvement of catalytic performance by providing more active oxygen species.

### 3.5. Scanning Electron Microscopy (SEM) and Transmission Electron Microscopy (TEM) Characterization

**Figure 6:**
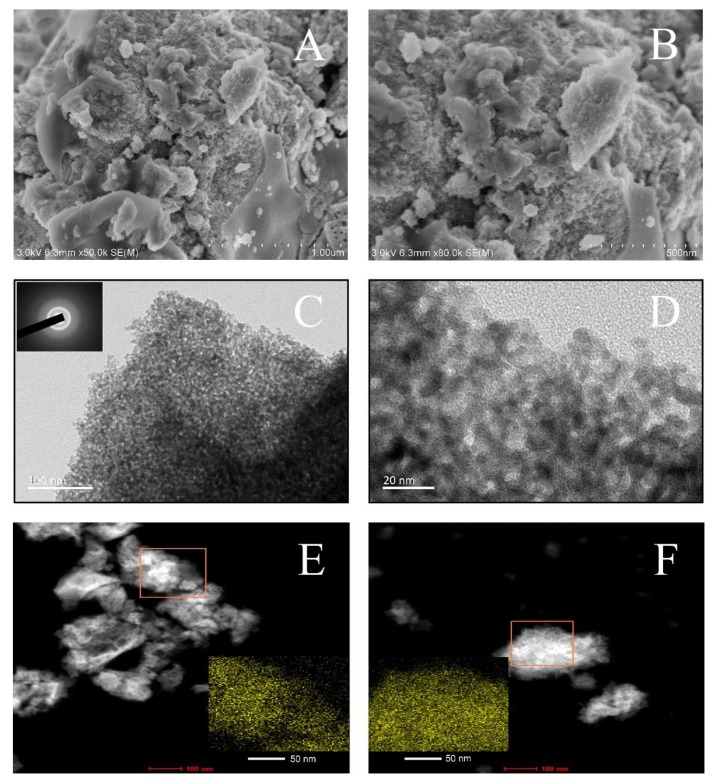
Scanning electron microscope (SEM) (**A**,**B**) and transmission electron microscope (TEM) (**C**,**D**) images of MCZ-0.67 catalyst, the inset is the pattern of the selected area electron diffraction (SAED). Scanning transmission electron microscope (STEM) images of MCZ-0.33 (**E**) and MCZ-0.67 (**F**) catalysts, the insets are Mn-element mappings.

The correlation between the catalytic performance and morphological characteristics of the promising MCZ-0.67 catalyst was studied using SEM and TEM. Figure 6A,B showed that the MCZ-0.67 catalyst presented sponge-like surface with a large degree of porosity, in agreement with its large BET. Closer observation using TEM (Figure 6C,D) showed that this catalyst was composed of well-distributed nanoparticles with a size of 6–10 nm, and their accumulation led to the formation of mesopores with a size of 3–9 nm. This morphology could contribute to the good catalytic performance to some extent by providing a large quantity of adsorption sites [45]. It is reported that nanoparticles showed promising applications for catalytic reactions due to their high surface-to-volume ratio [46]. The selected area electron diffraction (SAED) pattern exhibited in the inset further confirmed that the prepared MCZ-0.67 catalyst is amorphous since no typical diffraction rings or spot patterns were observed [47], in agreement with the results of the XRD characterization. The bright squares in Figure 6E,F indicated that the concentration and distribution of Mn species in MCZ-0.67 catalyst were better than those in MCZ-0.33 catalyst. As reported, the content and distribution of active Mn species can greatly influence the performance of the catalyst [48].

### 3.6. Catalytic Activity for Chlorobenzene (CB) Combustion

**Figure 7:**
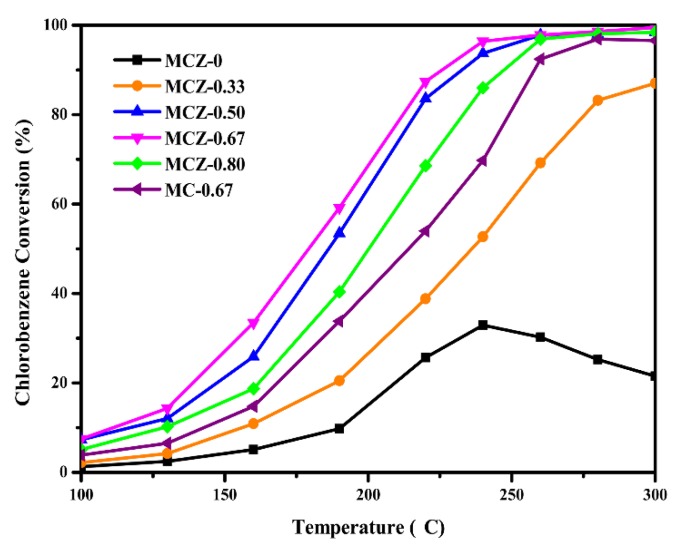
The catalytic activity of MCZ-x (x = 0, 0.33, 0.50, 0.67, 0.80) and MC-0.67 catalysts for chlorobenzene (CB) combustion; gas composition: 1000 ppm CB, 21% O_2_; gas hourly space velocity (GHSV) = 20,000 h^−1^.

The catalytic activity of the prepared catalysts during CB combustion was assessed (Figure 7 and Table 3). The MCZ-0 catalyst exhibited the lowest catalytic activity, with deactivation occurring after 250 °C. Following Mn doping, the catalytic activity and anti-poisoning ability were significantly improved. Specifically, MCZ-0.33 (Rs = 0.598 μmol m^−2^ h^−1^) exhibited an increased reaction rate compared to that of MCZ-0 (Rs = 0.468 μmol m^−2^ h^−1^) at 130 °C. This increase in reaction rate continued until x = 0.67 (Rs = 0.716 μmol m^−2^ h^−1^), after which a reversal was observed. Thus, the most abundant active sites may be generated on the MCZ-0.67 catalyst surface, as evidenced from Figure 6 that more active Mn species were evenly distributed in the catalyst. Therefore, the optimal Mn content for CB elimination was defined at 0.67.

The MCZ-0.67 catalyst achieved 90% CB conversion at approximately 226 °C (Table 3), which is lower than most transition metal oxide catalysts reported in previous literature (Table 4). Furthermore, this temperature was approximately 32 °C lower compared with the T_90_ of the MC-0.67 catalyst, likely due to the changes in physicochemical properties of the MC-0.67 catalyst following Zr doping, including the increase in BET surface area and active oxygen species. Additionally, the measurement of CO_2_ and CO concentrations in the off-gas (Table 3) showed that the prepared MCZ-x catalysts, except for the MCZ-0 catalyst, presented a greater than 98% CO_2_ selectivity, indicating that these catalysts possess a good deep catalytic oxidation of CB.

### 3.7. Catalyst Stability

Stability is another important consideration in the evaluation of catalytic performance, especially for the removal of CVOCs. Generally, dissociated chlorine species can occupy the catalyst active sites through adsorption or metal chloride formation, resulting in a decline in activity and stability, commonly known as chlorine poisoning [1]. Additionally, due to the strong electronegativity, chlorine deposited on the catalyst surface will lead to an electron shift towards the chlorine species [29], inhibiting the generation of active oxygen species. Accordingly, the timely removal of chlorine species from the catalyst surface is crucial for maintaining a stable catalytic activity.

**Figure 8:**
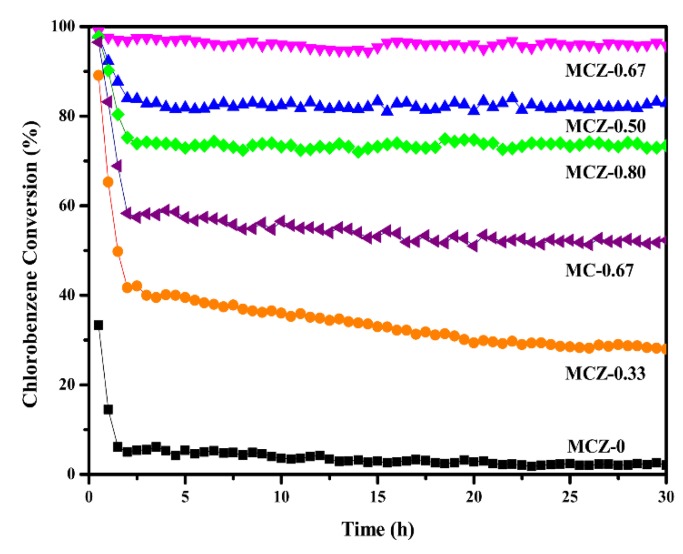
Stability tests of MCZ-x (x = 0, 0.33, 0.50, 0.67, 0.80) and MC-0.67 catalysts for CB elimination during 30 h of continuous reaction at 300 °C, gas composition: 1000 ppm CB, 21% O_2_; GHSV = 20,000 h^−1^.

The variation in catalytic activity over time at 300 °C for 30 h was assessed herein (Figure 8). The activity of the prepared catalysts decreased to varying degrees within the initial 2.5 h likely due to the rapid deposition of chlorine species on the active sites. The activity of the MCZ-0 catalyst was largely exhausted at the end of the stability test, mainly due to its poor redox properties (revealed in TPR characterization). With the introduction of Mn, the increased BET surface area and surface active oxygen could significantly improve the catalyst chlorine resistance by providing more active sites and accelerating the removal of deposited chlorine. It is worth noting that the MCZ-0.67 catalyst maintained a high and stable catalytic activity (>95%) and showed the best resistance to chlorine poisoning throughout the stability test. In contrast, the MC-0.67 catalyst showed a significant decrease in catalytic activity, from 96% to 52%, after 30 h of continuous reaction. Therefore, the addition of Zr led to an improvement in catalytic stability.

**Figure 9:**
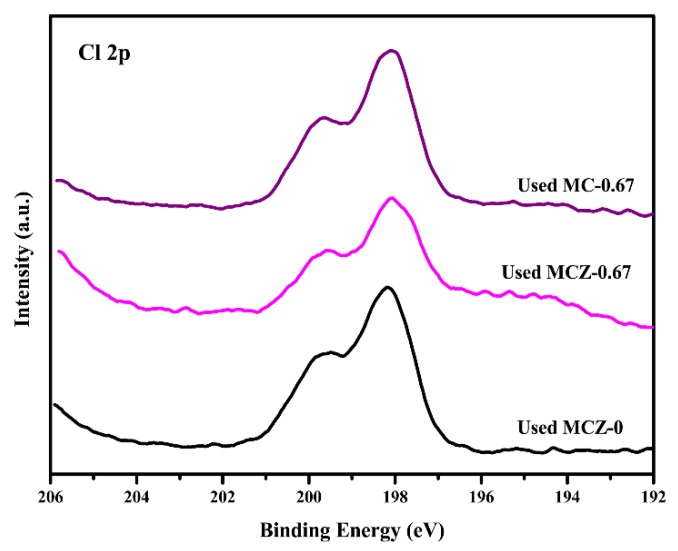
Cl 2p XPS spectra of the used MCZ-0, MCZ-0.67 and MC-0.67 catalysts.

Chlorine resistance was assessed according to the Cl 2p XPS spectra of the used MCZ-0, MCZ-0.67 and MC-0.67 catalysts after the stability tests (Figure 9). The two overlapping peaks appearing at approximately 198.1 eV and 199.6 eV were attributed to the Cl 2p_3/2_ and Cl 2p_1/2_ core levels, respectively [54]. Their presence confirmed the occurrence of chlorine deposition on the catalyst surface. Specifically, according to the estimation from the peak area of Cl 2p_3/2_, the surface chlorine content of the used MCZ-0, MCZ-0.67 and MC-0.67 catalysts was 5.9 at%, 4.0 at% and 5.2 at%, respectively. Obviously, the MCZ-0.67 catalyst had the best chlorine resistance, with Zr addition effectively inhibiting chlorine poisoning.

**Figure 10:**
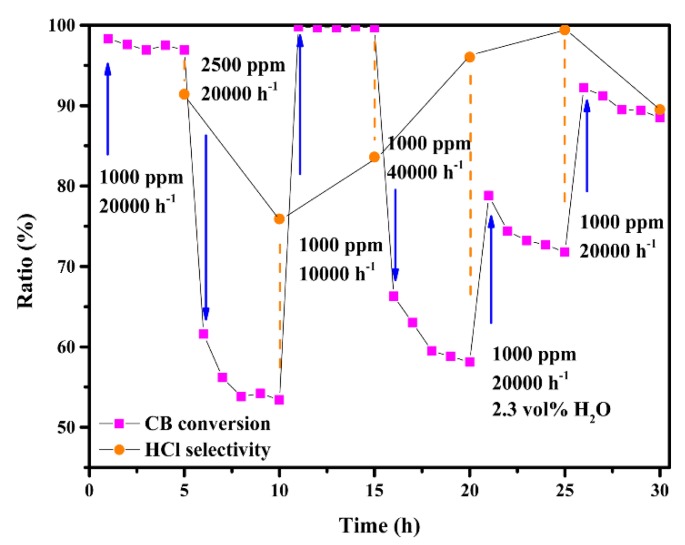
The successive influence of CB concentration, space velocity and water vapor on CB conversion and HCl selectivity over the MCZ-0.67 catalyst at 300 °C.

Apart from time-based stability, the practical application process subjects catalysts to complicated and variable operating conditions. Therefore, the successive influence of CB concentration, space velocity and water vapor on CB elimination and HCl selectivity ([HCl]/([HCl] + [Cl_2_])) were studied for the MCZ-0.67 catalyst at 300 °C (Figure 10). Both higher CB concentration (2500 ppm) and space velocity (40,000 h^−1^) led to a serious decrease in activity (lower than 60%), while lower space velocity (10,000 h^−1^) resulted in an almost complete elimination of CB. The influence of CB concentration indicated that the catalytic efficiency would be overloaded when handling a large amount of reactants, and the influence of lower space velocity was attributed to a more sufficient oxidation caused by the longer residence time of reactants through the catalyst bed. Meanwhile, Deacon reaction (the generation of Cl_2_) was similarly influenced, as can be seen from the higher HCl selectivity at greater space velocity. Under water vapor condition (2.3 vol%), the conversion of CB dropped to 72%, which is attributed to the competitive adsorption of CB and H_2_O on the surface active sites [55]. In contrast, the selectivity of HCl was as high as 99%, showing that water can effectively inhibit the generation of Cl_2_. According to previous report, the flowing water vapor can wash the catalyst surface and promote the formation of HCl by the dissociated chlorine species [56]. Thus, as shown above, despite undergoing various operating conditions, CB conversion was maintained at approximately 90%, further confirming the good catalytic performance of the MCZ-0.67 catalyst.

### 3.8. Regeneration of Catalyst

**Figure 11:**
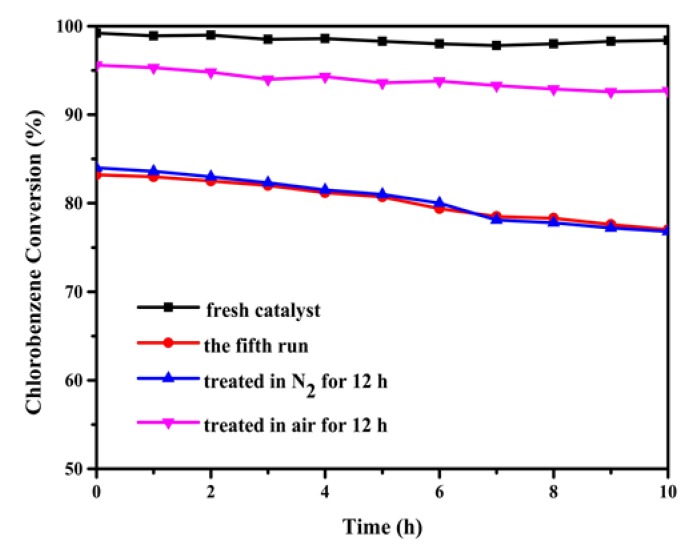
The catalytic efficiency of the MCZ-0.67 catalyst treated under different conditions; activity test condition: 300 °C for 10 h; gas composition: 1000 ppm CB, 21% O_2_; GHSV = 20,000 h^−1^.

Catalytic combustion is a heterogeneous catalytic oxidation process, varying from homogeneous catalysis, catalysts used in the tests can be easily separated from the reaction system [57]. Thus, it is of great significance to explore the reuse of catalysts (Figure 11). After the fourth reaction run, MCZ-0.67 catalyst suffers a decrease in catalytic efficiency from 98% (fresh catalyst) to approximately 80% at 300 °C. Regeneration was carried out by calcining the above used catalyst at 350 °C for 12 h in air and nitrogen atmosphere respectively. As shown in Figure 11, after treatment in air, the activity of MCZ-0.67 catalyst recovered to approximately 95%, while that of the catalyst treated in nitrogen remained almost unchanged. This phenomenon indicates that the presence of oxygen contributes to the regeneration of the catalyst and the used MCZ-0.67 catalyst can be recycled by calcination in air. The re-enhancement of catalytic activity may be attributed to the reaction of oxygen species with chlorine species at high temperature, which exposes the active sites occupied by chlorine species again [11].

During the above 10 h of continuous test, all the off-gas was bubbled through a 5% HNO_3_ solution. After concentration, inductively coupled plasma–atomic emission spectroscopy (ICP–AES, Thermo Scientific 6300) was used to measure the metal elements in the solution. Results showed that the corresponding Mn, Ce and Zr elements were not detected (detection limit is 0.01 ppm), indicating that the prepared MCZ-0.67 catalyst was stable in composition, and no metal elements were leached into the final effluents under this reaction media.

### 3.9. Product Analysis 

**Figure 12:**
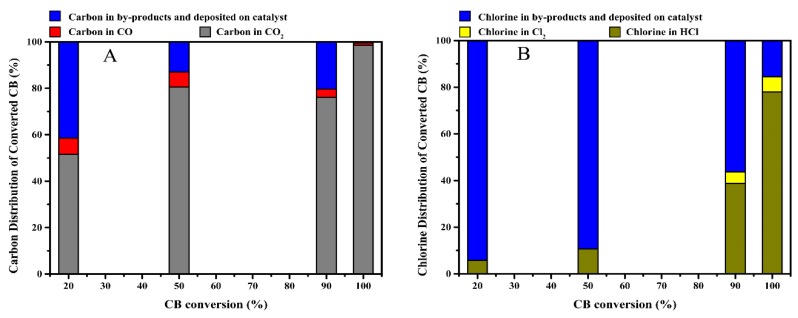
Carbon distribution (**A**) and chlorine distribution (**B**) of converted CB at different conversion rates over the MCZ-0.67 catalyst.

In order to better elucidate the CB oxidation process, the CB decomposition products in the off-gas were collected and analyzed. Based on the calculation of carbon balance and chlorine balance of reactants, the carbon distribution and chlorine distribution of converted CB at different conversion rates (i.e., 20%, 50%, 90% and 100% CB conversion, the corresponding temperature was at approximately 145, 185, 235 and 300 °C, respectively) over the MCZ-0.67 catalyst were obtained (Figure 12). The carbon in the decomposed CB mainly existed in the form of CO_2_, with CO_2_ selectivity increasing with the increase in catalytic activity (Figure 12A). When CB was completely oxidized, the selectivity of carbon to carbon oxides (CO_2_ and CO) exceeded 99.5%, of which the CO content was less than 1%. However, the selectivity of chlorine to HCl in converted CB was low at 20% and 50% CB conversion (Figure 12B), likely related to the easier adsorption of chlorine species on the catalyst surface at lower temperatures [12]. Furthermore, Cl_2_ was detected at 90% CB conversion, indicating the occurrence of the Deacon reaction. Indeed, it has been previously reported that the higher temperature and modified Mn species are both favorable for the production of Cl_2_ [49]. Cl balance gives the calculation result of 84% at 100% CB conversion, combined with the high carbon oxides selectivity at this time, the occurrence of chlorine deposition on catalyst surface can be confirmed, which may be related to the formation of metal chlorides or metal oxychlorides (MCl_x_ or MOCl_x_).

**Figure 13:**
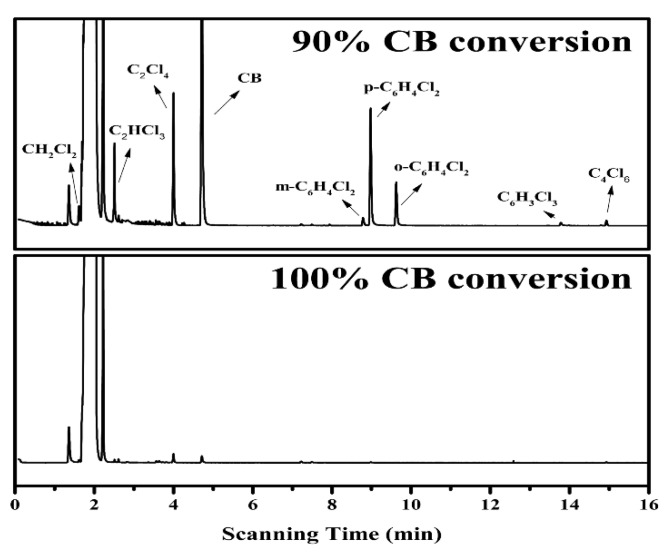
The gas chromatography-mass spectrometer (GC-MS) chromatograms of organic by-products in off-gas at different CB conversion rates over the MCZ-0.67 catalyst.

**Scheme 1:**
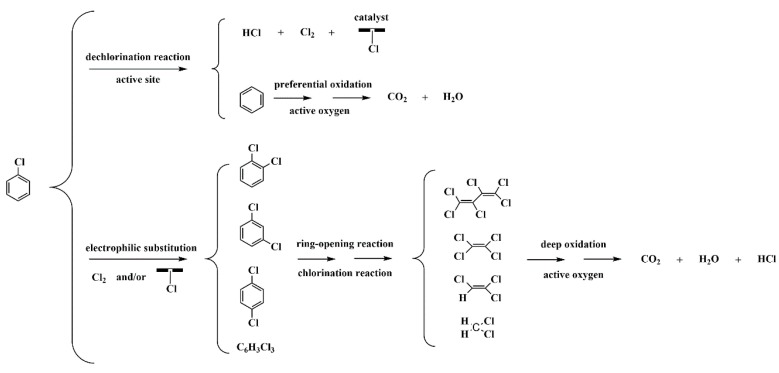
Proposed reaction path of CB oxidation over the MCZ-0.67 catalyst.

The production of polychlorinated by-products is inevitable during the oxidation of chlorinated aromatic hydrocarbons due to the strong reactivity of chlorine species. Thus, an evaluation of the CB decomposition products is required. Herein, the GC-MS method was employed for the detection of probable trace products (Figure 13), showing that various polychlorinated by-products were produced at 90% CB conversion, including dichlorobenzene (C_6_H_4_Cl_2_, ortho—6 ppm, meta—2 ppm, para—16 ppm), tetrachloroethylene (C_2_Cl_4_, 15 ppm), trichloroethylene (C_2_HCl_3_, 7 ppm), dichloromethane (CH_2_Cl_2_, 3 ppm) and trace amounts of trichlorobenzene (C_6_H_3_Cl_3_, <1 ppm) and hexachlorobutadiene (C_4_Cl_6_, <1 ppm). Their presence directly confirms the occurrence of chlorination reactions during CB oxidation and clearly depicts the break-down process of the C–C bond from a hexatomic ring to a short chain. It has been previously reported that the formation of these polychlorinated by-products is initiated from the electrophilic substitution of chlorine species [55], and according to the chlorine distribution during CB oxidation (Figure 12B), it can be reasonably speculated that the chlorine species dissociated from the catalyst surface and the Cl_2_ resulting from the Deacon reaction are responsible for the substitution reaction. In addition, it is well acknowledged that the destruction of CB molecule begins with dechlorination reaction due to the lower dissociation energy of the C–Cl bond than that of C–H bond [49], and the resulting intermediate benzene is then further ring-opened and oxidized by active oxygen species. Herein, among the products of CB decomposition, benzene, the incomplete oxidation product of CB, was not detected, while it is the only organic by-product during the oxidation of CB over Mn_0.8_Ce_0.2_O_2_ catalyst [55]. This phenomenon may be ascribed to the competitive adsorption for the same active sites between polychlorobenzene and benzene; herein, benzene seems to be preferentially oxidized over the MCZ-0.67 catalyst. This result may be correlated to the higher electron cloud density of benzene than that of polychlorobenzene, thus facilitating the formation of π-complex in the adsorption process [52]. As the reaction temperature increases, these small amounts of polychlorinated by-products can be further oxidized under the strong oxidation ability of active oxygen species, as evidenced from the observations in Figure 13, wherein the corresponding peaks are almost invisible at 100% CB conversion. Based on the above analyses, a possible reaction path of CB oxidation over the MCZ-0.67 catalyst was proposed and presented in Scheme 1.

## 4. Conclusions

Mn-Ce-Zr-O catalysts doped with a varying Mn concentration were prepared and assessed for the catalytic combustion of CB. Nanosized MCZ-0.67 catalyst with amorphous phase was found to exhibit superior catalytic activity and chlorine resistance among the studied catalysts, and presented excellent performance in stability tests, including time-based stability and the successive influence of the various operating conditions. At the same time, the used MCZ-0.67 catalyst can be recycled by calcination in air at 350 °C for 12 h. Characterization results showed that the doping of Mn played a dominant role in improving the catalytic performance by increasing the BET surface area, improving the redox properties and increasing the percentage of surface active oxygen. Product analysis showed that trace amounts of polychlorinated by-products did occur during CB oxidation, attributed to the chlorination reactions caused by the dissociated chlorine species and the Cl_2_ generated. At 300 °C, these by-products can be completely oxidized and a high CO_2_ selectivity (>99%) was obtained under the strong oxidation ability of the MCZ-0.67 catalyst. Therefore, the MCZ-0.67 catalyst prepared herein shows promise for applications to eliminate CVOCs. In addition, although ZrO_2_ is inactive, the changes in physical structure and chemical properties of catalysts resulting from Zr introduction are essential for maintaining their high and stable catalytic activity.

## Figures and Tables

**Table 1:** XRD, N_2_ adsorption-desorption and H_2_-temperature programmed reduction (H_2_-TPR) characterization data.

Catalyst	Lattice Parameter (Å) ^1^	Crystallite Size (nm) ^2^	Brunauer–Emmett–Teller Surface Area (m^2^ g^−1^)	Pore Volume (cm^3^ g^−1^)	Average Pore Size (nm)	H_2_ Consumption (mmol g^−1^)
MCZ-0	5.32	6.5	35.7	0.06	5.8	0.834
MCZ-0.33	5.29	3.7	47.0	0.09	6.4	2.242
MCZ-0.50	5.35	2.2	116.9	0.11	3.2	3.431
MCZ-0.67	–	–	135.6	0.19	4.0	5.081
MCZ-0.80	–	–	101.1	0.16	4.8	6.151
MC-0.67	5.36	3.5	67.6	0.12	5.7	4.334

^1^ Calculated from the CeO_2_ crystalline phase; ^2^ The value estimated by Scherrer equation from the (111) reflection of CeO_2_.

**Table 2:** XPS results of MCZ-x (x = 0, 0.33, 0.50, 0.67, 0.80) and MC-0.67 catalysts.

Catalyst	Mn/(Mn + Ce + Zr) Molar Ratio	Ce^3+^/(Ce^3+^ + Ce^4+^) (%)	Mn^4+^/(Mn^3+^ + Mn^4+^) (%)	O_β_ /(O_α_ + O_β_ + O_γ_) (%)
MCZ-0	0	28.6	–	25.5
MCZ-0.33	0.41	30.5	40.6	29.3
MCZ-0.50	0.54	32.5	41.3	41.3
MCZ-0.67	0.70	34.3	42.5	47.7
MCZ-0.80	0.81	31.8	37.8	36.0
MC-0.67	0.72	31.3	40.7	31.5

**Table 3:** Results of activity tests on CB combustion over the prepared catalysts.

Catalyst	T_90_ (°C) ^1^	S_CO2_ (%) ^2^	Rs at 130 °C (μmol m^−2^ h^−1^) ^3^
MCZ-0	>300	86.3	0.468
MCZ-0.33	>300	98.4	0.598
MCZ-0.50	233	98.9	0.693
MCZ-0.67	226	99.2	0.716
MCZ-0.80	248	99.0	0.675
MC-0.67	258	97.7	0.644

^1^ The temperature at which CB conversion reaches 90%. ^2^ CO_2_ selectivity measured at 300 °C and calculated based on formula: S_CO2_ = [CO_2_]/([CO_2_] + [CO]). ^3^ Reaction rate of CB oxidation normalized by catalyst specific surface area.

**Table 4:** Main data of literature reports on CB elimination over transition metal oxide catalysts.

Catalyst	Concentration (ppm)	GHSV (h^−1^)	T_90_ (°C)	Refs.
4Ce1Cr	1000	15,000	260	[7]
MnO_x_ (0.86)-CeO_2_	1000	15,000	~240	[11]
10%Sn-Mn-Ti	500	20,000	177	[32]
Mn (0.86)-CeLa	1000	15,000	279	[49]
CuMnCeO_x_	600	30,000	255	[50]
La_0.8_Sr_0.2_MnO_3_	1000	15,000	291	[51]
2.1% VO_x_/CeO_2_	1000	30,000	~308	[52]
Mn_8_Co_1_Ce_1_/cordierite	500	15,000	325	[12]
Mn-Ce-Mg/Al_2_O_3_	1000	15,000	315	[53]
MCZ-0.67	1000	20,000	226	This work
